# Psychotic depression associated with Graves' disease: A case report on treatment prioritization

**DOI:** 10.1002/pcn5.70419

**Published:** 2026-09-30

**Authors:** Ayumu Kocha, Aya Akaba, Nozomi Sawasaki, Shinobu Seno, Naoya Hara, Taro Minato, Atsuo Nakagawa, Hiroki Kocha

**Affiliations:** ^1^ Department of Neuropsychiatry St. Marianna University School of Medicine Kawasaki Kanagawa Japan; ^2^ Department of Psychiatry Sakuragaoka Memorial Hospital Tama Tokyo Japan

**Keywords:** endogenous psychosis, Graves' disease, modified electroconvulsive therapy, psychotic depression, symptomatic psychosis

## Abstract

**Background:**

Graves' disease can be accompanied by psychiatric symptoms, including affective and psychotic manifestations. When a well‐defined psychiatric syndrome occurs concurrently with a medical illness, determining whether it is directly attributable to the medical condition or represents an independent psychiatric disorder precipitated by it can be difficult. Treatment sequencing becomes particularly important when psychiatric symptoms interfere with treatment of the medical illness.

**Case Presentation:**

A woman in her 50s was admitted with severe psychotic depression characterized by delusions of guilt, hypochondriacal and poisoning delusions, marked agitation, food refusal, and medication refusal. Graves' disease was diagnosed concurrently. Severe agitation prevented adequate nutritional support and initiation of antithyroid treatment, and intravenous haloperidol produced little improvement. After confirming hemodynamic stability and the absence of clinical features suggestive of thyroid storm, modified electroconvulsive therapy (mECT) was initiated in consultation with endocrinology and anesthesiology. Agitation markedly improved after the first session, allowing physical restraint to be discontinued and oral treatment to begin. Methimazole was subsequently initiated. The GRID‐HAMD‐17 score was 39 on hospital Day 11 and 2 on hospital Day 36. Six mECT sessions were administered, and psychiatric remission was maintained during 2 months of outpatient follow‐up.

**Conclusion:**

The etiological relationship between Graves' disease and psychotic depression cannot be determined from temporal association alone. Nevertheless, treatment decisions cannot always await etiological clarification. When severe psychiatric symptoms prevent treatment of a coexisting medical illness, rapid psychiatric stabilization may itself be necessary to enable medical treatment. In this case, mECT rapidly improved severe psychomotor agitation and behavioral dysregulation, thereby restoring the feasibility of treating the coexisting physical illness.

## BACKGROUND

Psychiatric manifestations associated with physical illness have long been recognized. Bonhoeffer (1910)^1^ described common psychiatric manifestations arising from different physical illnesses and later (1917) proposed the concept of “acute exogenous reaction types.”^2^ Bleuler (1954) described psychiatric changes associated with endocrine disorders as “endocrine psychosyndromes,”^3^ while Wieck (1956) described psychiatric syndromes without overt disturbance of consciousness as “transitional syndromes.”^4^ These concepts highlight that somatic etiology and psychopathological presentation do not necessarily correspond in a one‐to‐one manner.

Graves' disease and thyrotoxicosis can be associated with a broad range of psychiatric manifestations, including anxiety, mood symptoms, agitation, delirium, and, less commonly, psychosis.^5^, ^6^, ^7^, ^8^, ^9^, ^10^, ^11^ When a syndrome resembling a primary psychiatric disorder occurs concurrently with Graves' disease, it may be difficult to determine whether the syndrome is directly attributable to thyroid dysfunction or whether the medical illness precipitated an otherwise independent psychiatric episode.

This etiological distinction is important but may not resolve an immediate clinical problem. Severe psychiatric symptoms can interfere with examination, nutrition, medication, and treatment of the underlying medical condition. In such circumstances, clinicians must determine treatment priorities even when causality remains uncertain.

We report a woman with psychotic depression and Graves' disease whose severe agitation, food refusal, and medication refusal initially prevented treatment of hyperthyroidism. mECT rapidly improved her psychiatric condition and enabled treatment of the physical illness. We discuss the case from the perspectives of a psychiatric syndrome directly attributable to Graves' disease versus an endogenous psychosis precipitated by it, with particular attention to treatment prioritization.

## CASE PRESENTATION

Written informed consent for publication of this case report, including clinical details and anonymized quotations, was obtained from the patient.

The patient was a woman in her 50s. More than 20 years previously, she had undergone right thyroid lobectomy, although the indication was unknown. Details of any previous thyroid hormone replacement therapy were unavailable; however, she was not receiving thyroid hormone replacement at the time of admission. She had no previous psychiatric treatment, relevant family psychiatric history, or history of alcohol or psychoactive substance misuse. Development was unremarkable. After nursing school, she worked as a nurse and lived with her junior‐high‐school‐aged daughter. Premorbidly, she was cheerful and sociable but highly conscientious, meticulous, and considerate of others.

In March of year X, at age 50, she was transferred to another department at work. She became increasingly concerned that she could not perform her new duties adequately and developed insomnia. By mid‐May she had become increasingly restless, and by late May she had difficulty attending work. On June 2, she told colleagues that she was “not needed” and would be dismissed. At the Department of Neurology at Hospital A, marked agitation was observed. Although tremor and tachycardia were absent, laboratory testing demonstrated suppressed thyroid‐stimulating hormone (TSH) and elevated free triiodothyronine (FT3) and free thyroxine (FT4). Her oral intake subsequently decreased, with a 3.5‐kg weight loss over 1 month.

On June 4, she was admitted to the Department of Internal Medicine at Hospital B for evaluation of hyperthyroidism. Because severe agitation and unpredictable behavior precluded adequate medical treatment, she was transferred to our psychiatric hospital the following day.

On admission, she repeatedly stated, “It is my fault” and “I have done something terrible.” She also believed that her body emitted a strong odor and that her food and intravenous fluids contained poison, consistent with delusions of guilt, hypochondriacal delusions, and delusions of poisoning. Depressed mood, loss of motivation, severe psychomotor agitation, and restlessness were prominent. Orientation was preserved, although communication was difficult because of severe agitation. No clear disturbances of self‐experience were identified. Neurological examination was unremarkable, and tremor and marked tachycardia were absent. The psychiatric presentation was considered a severe depressive syndrome with psychotic features rather than delirium.

Laboratory testing showed TSH 0.006 μIU/mL, FT4 2.48 ng/dL, FT3 4.65 pg/mL, thyroid‐stimulating antibody 212%, and thyrotropin receptor antibody 4.6 IU/L. Thyroid ultrasonography performed at the referring hospital showed enlargement of the left lobe. Noncontrast head computed tomography revealed no acute intracranial abnormality.

On admission, she refused all oral medications, including both scheduled and as‐needed psychotropic medications. While her physical condition was being assessed, oral treatment was attempted first, and pharmacologic sedation was not initiated immediately. Seclusion was initiated on hospital Day 2. Because food and fluid intake remained inadequate, intravenous therapy was attempted; however, severe agitation and resistance necessitated physical restraint, and nasogastric feeding could not be safely implemented. Intravenous haloperidol 10 mg/day was initiated on hospital Day 6 but produced little improvement.

Because rapid psychiatric stabilization was considered necessary to permit treatment of the medical condition, a decision was made to initiate mECT using brief‐pulse stimulation on hospital Day 8 after consultation with endocrinology and anesthesiology regarding the risks associated with hyperthyroidism. An electrocardiogram obtained on admission showed no clinically significant abnormalities. On admission, her blood pressure was 136/97 mmHg, pulse rate was 85 beats/min, body temperature was 36.7°C, and she was fully conscious. Transthoracic echocardiography was considered but could not be performed because of severe agitation and resistance. Vital signs remained stable from admission to the first mECT session, without sustained tachycardia, fever, or hemodynamic instability. Immediately before the first mECT session, her blood pressure was 128/85 mmHg, pulse rate was 83 beats/min, and body temperature was 37.1°C. There was no disturbance of consciousness or clinical evidence of heart failure, and thyroid storm was not clinically suspected. mECT was performed using a Thymatron® system (Somatics Inc., Lake Bluff, IL, USA) with bitemporal electrode placement. Anesthesia was induced with propofol 50 mg, and neuromuscular blockade was achieved with suxamethonium 40 mg. At the first session on hospital Day 8, the stimulus intensity was 25%, with a delivered charge of 125 mC, pulse width of 0.5 ms, frequency of 20 Hz, and stimulus duration of 7 s. The electroencephalographic seizure duration was approximately 40–45 s. No motor seizure was observed, and seizure adequacy was not clearly established. On return to the ward after the first mECT session, her blood pressure was 125/87 mmHg, pulse rate was 85 beats/min, and body temperature was 37.0°C. She was initially drowsy following mECT but gradually became fully alert. No clinically significant arrhythmia, sustained tachycardia, marked hypertensive response, fever, or other adverse event was observed. After the first mECT session, agitation and hyperactivity subsided sufficiently for physical restraint to be discontinued on hospital Day 9. Because the thyroid scintigraphy results were not yet available and destructive thyroiditis could not be excluded, propranolol was initiated as temporary symptomatic treatment pending etiological clarification.

On hospital Day 11, oral psychotropic medication was accepted; intravenous haloperidol was discontinued, and olanzapine 10 mg/day and mirtazapine 15 mg/day were initiated. The second mECT session was also performed on hospital Day 11. Because seizure adequacy had not been clearly established during the first session, the stimulus intensity was increased to 40%, with a delivered charge of 201–203 mC, pulse width of 0.5 ms, frequency of 30 Hz, and stimulus duration of 7.5 s. From the second session onward, electroencephalographic seizure duration was approximately 40–45 s and motor seizure duration was approximately 20–30 s, with adequate seizures consistently obtained.

Methimazole had been planned if Graves' disease was confirmed. The thyroid scintigraphy results subsequently became available, showing clear tracer uptake in the remaining left thyroid lobe, with an uptake rate of 1.74% and without focal or nodular uptake. These findings, together with the positive thyroid autoantibodies, supported the diagnosis of Graves' disease rather than thyroiditis or a functional thyroid nodule, and methimazole was initiated on hospital Day 12. Further mECT sessions were administered on hospital Days 15 and 18. Her depressive symptoms and delusions subsequently subsided, and seclusion was discontinued on hospital Day 19.

Additional mECT sessions were administered on hospital Days 21 and 25, completing a total of six sessions. Electrocardiography, noninvasive blood pressure, and pulse oximetry were continuously monitored during each procedure, and no cardiovascular or other clinically significant adverse events occurred during the mECT course.

Her GRID‐HAMD‐17 score was 39 on hospital Day 11 and 2 on hospital Day 36. The temporal relationship between psychiatric symptoms, FT4 levels, and major treatment events is shown in Figure 1. She was discharged home on hospital Day 39 while receiving mirtazapine 15 mg/day and olanzapine 10 mg/day. At the 2‐month outpatient follow‐up, olanzapine had been discontinued, and mirtazapine 15 mg/day was continued. Methimazole 5 mg once weekly was also continued. She remained free of psychotic and depressive symptoms. Maintenance ECT had been considered but was not administered. Thyroid function at the 2‐month follow‐up was TSH 0.331 μIU/mL, FT3 2.38 pg/mL, and FT4 1.08 ng/dL. The clinical course is summarized in Tables 1, 2, 3.

**Figure 1:**
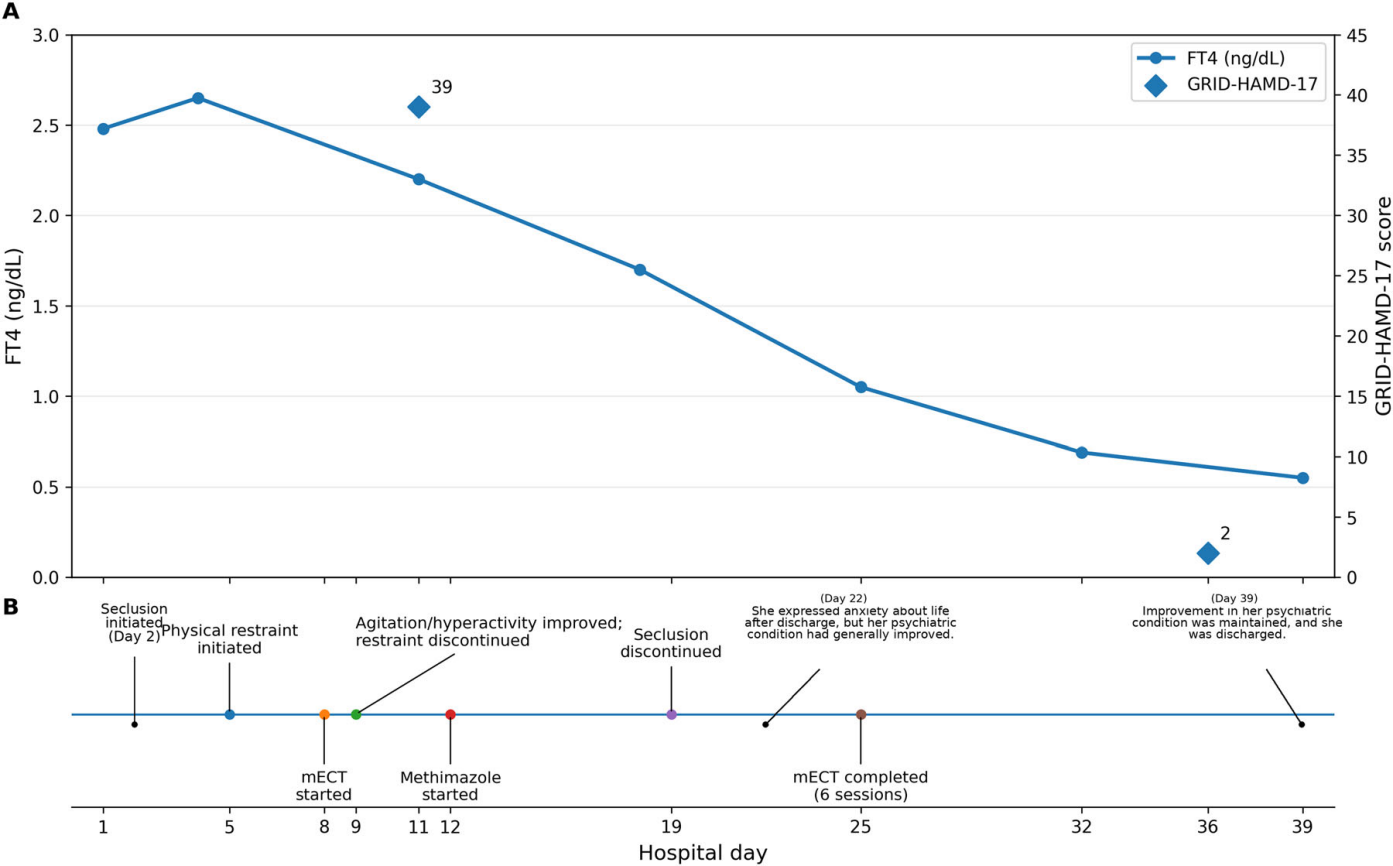
Clinical course of psychiatric symptoms, thyroid function, and treatment. (A) Serial changes in FT4 levels and GRID‐HAMD‐17 scores. (B) Timeline of major clinical events and treatment interventions. Serial FT4 values are shown on the left axis. GRID‐HAMD‐17 scores are shown as individual points on the right axis and were assessed on hospital Days 11 and 36; the score at admission could not be obtained because of severe agitation. Key clinical events and treatment interventions are shown below. FT4, free thyroxine; GRID‐HAMD‐17, 17‐item GRID version of the Hamilton Depression Rating Scale; mECT, modified electroconvulsive therapy.

**Table 1:** Changes in thyroid function test results after admission.

Time point	TSH (μIU/mL)	FT3 (pg/mL)	FT4 (ng/dL)	TRAb (IU/L)
On admission/hospital Day 1	0.006	4.65	2.48	4.6
Hospital Day 4	0.008	4.40	2.65	Not measured
Hospital Day 11	<0.005	3.49	2.20	Not measured
Hospital Day 18	<0.005	3.23	1.70	Not measured
Hospital Day 25	<0.005	2.94	1.05	Not measured
Hospital Day 32	0.016	2.73	0.69	Not measured
Hospital Day 39	0.067	2.16	0.55	Not measured
Approximately 1 month after discharge	0.606	2.25	0.79	Not measured
Approximately 2 months after discharge	0.331	2.38	1.08	Not measured

Abbreviations: FT3, free triiodothyronine; FT4, free thyroxine; TRAb, thyrotropin receptor antibody; TSH, thyroid‐stimulating hormone.

**Table 2:** Clinical course and changes in psychiatric treatment and treatment for Graves' disease/hyperthyroidism.

Time point	Clinical course	Psychiatric treatment	Treatment for Graves' disease/hyperthyroidism
On admission/hospital Day 1	Marked agitation was present. Delusions of guilt, hypochondriacal delusions, and delusions of poisoning were prominent.	[*]	[*]
Hospital Day 2	Marked agitation and hyperactivity were observed, and seclusion was initiated.	[*]	[*]
Hospital Day 3	—	[*]	[*]
Hospital Day 5	Marked agitation and hyperactivity persisted, and physical restraint was initiated.	[*]	[*]
Hospital Day 6	—	Intravenous HPD 10 mg/day was initiated.	[*]
Hospital Day 8	—	Modified electroconvulsive therapy (mECT) was initiated.	[*]
Hospital Day 9	Marked agitation and hyperactivity subsided, and physical restraint was discontinued.	[*]	PRO 30 mg/day was initiated.
Hospital Day 11	Although diurnal variation persisted, the depressive delusions showed a tendency to resolve.	Intravenous HPD 10 mg/day was discontinued, and oral OLZ 10 mg/day and MTZ 15 mg/day were initiated.	—
Hospital Day 12	—	—	MMI 10 mg/day was initiated.
Hospital Day 18	—	—	MMI was reduced to 5 mg/day.
Hospital Day 19	Agitation improved, and seclusion was discontinued.	—	—
Hospital Day 22	She expressed anxiety about life after discharge, but her psychiatric condition had generally improved.	—	MMI was changed to 5 mg every other day, and PRO was discontinued.
Hospital Day 25	—	A total of six mECT sessions had been administered, and the course of mECT was completed.	—
Hospital Day 39	Improvement in her psychiatric condition was maintained, and she was discharged.	—	—

*Note*: “—” indicates that there were no noteworthy events at the corresponding time point. “*” indicates refusal of medication.

Abbreviations: HPD, haloperidol; mECT, modified electroconvulsive therapy; MMI, methimazole (thiamazole); MTZ, mirtazapine; OLZ, olanzapine; PRO, propranolol.

**Table 3:** Changes in GRID version of the Hamilton Depression Rating Scale (GRID‐HAMD) scores.

Time point	GRID‐HAMD‐17	GRID‐HAMD‐21
On admission/hospital Day 1	Not assessable^a^	Not assessable^a^
Hospital Day 11	39	41
Hospital Day 36	2	2

^a^ Assessment was not possible because of marked agitation.

## DISCUSSION

### Somatic and psychopathological diagnoses

Two diagnostic descriptions apply to this case: the somatic medical diagnosis of Graves' disease and the psychopathological diagnosis of psychotic depression. Graves' disease was established by thyroid function tests, thyroid autoantibodies, and thyroid scintigraphy, whereas psychotic depression was identified syndromically from depressed mood, psychomotor agitation, delusions of guilt, and hypochondriacal delusions. The central question is therefore not whether the two diagnoses coexist, but how they are related: whether the psychotic depression was a direct psychiatric manifestation of Graves' disease or whether Graves' disease precipitated an independent psychiatric disorder.

### Symptomatic or triggered?

The temporal association is important when assessing whether a psychiatric syndrome is attributable to a physical illness. Correspondence between changes in the medical illness and emergence or resolution of psychiatric symptoms supports a direct relationship, whereas persistence or recurrence of psychiatric symptoms after improvement of the medical illness may support an independently evolving psychiatric disorder. Neither pattern alone establishes causality. In this case, Graves' disease and psychiatric symptoms were identified at approximately the same time, and the available course does not allow the two possibilities to be distinguished definitively.

Notably, the acute psychiatric and endocrine courses were not entirely parallel. Marked agitation and hyperactivity improved rapidly after the initiation of mECT on hospital Day 8, allowing physical restraint to be discontinued on hospital Day 9, whereas methimazole was not initiated until hospital Day 12 and biochemical hyperthyroidism persisted at that time. This temporal dissociation may argue against a simple model in which the acute psychiatric state was driven solely by the severity of thyrotoxicosis. However, it does not exclude Graves' disease as a precipitating or contributing factor in the development of the psychiatric episode.

The DSM‐5‐TR similarly considers biological plausibility, temporal relationship, and typicality of presentation when evaluating whether a psychiatric disorder is attributable to another medical condition.^12^ In the present case, the patient did not exhibit a nonspecific mental state dominated by delirium or impaired consciousness, as may occur in thyroid storm,^5^ but rather with a relatively coherent syndrome of psychotic depression characterized by delusions of guilt, hypochondriacal delusions, and marked agitation.

Psychotic manifestations of thyrotoxicosis are uncommon, and presentations in which prominent psychiatric symptoms occur despite relatively mild classic somatic or adrenergic signs appear to be rare. The frequency of this particular presentation has not been well quantified, with the available literature consisting largely of case reports and small case series.^6^, ^7^, ^8^, ^9^, ^10^, ^11^

Birnbaum's structural analysis (*Strukturanalyse*) (1923) distinguished between pathogenetic factors, which contribute to the development of a psychopathological condition, and pathoplastic factors, which shape its particular clinical expression.^13^ Although the terminology is historical, the framework remains useful. Its value lies in moving beyond asking whether factors are associated with a syndrome to asking how each contributes to its particular form. In the present case, even if Graves' disease contributed to the development of the psychiatric syndrome, individual predisposition, personality, and life circumstances may have influenced why the resulting syndrome took the form of psychotic depression. Thus, the etiological question of whether the syndrome was directly attributable to Graves' disease or precipitated by it should be distinguished from the question of why this particular psychopathological presentation emerged.

### Treatment prioritization when physical illness coexists with severe psychiatric symptoms

Even when the etiological relationship between a psychiatric syndrome and a physical illness remains uncertain, treatment decisions cannot be deferred. Both conditions require consideration, and treatment sequencing should reflect not only severity and urgency but also the feasibility of treating the physical illness. Psychiatric symptoms may themselves compromise the patient's ability to cooperate with examination and treatment.

Based on previously reported cases,^6^, ^7^, ^8^, ^9^, ^10^, ^11^ these situations can be broadly organized into three patterns:

1. Treatment of the physical illness is feasible without active psychiatric treatment.
2. Treatment of the physical illness is feasible, and medical and psychiatric treatments can proceed concurrently.
3. Psychiatric symptoms interfere with treatment of the physical illness, making psychiatric stabilization necessary before medical treatment can proceed.

The present case corresponded to the third pattern. Marked agitation and restlessness, food refusal, and medication refusal made both psychiatric pharmacotherapy and treatment of Graves' disease difficult. Intravenous haloperidol produced little improvement, and rapid psychiatric stabilization was required to make treatment of the physical illness possible.

mECT in the presence of untreated hyperthyroidism requires careful medical assessment because the transient autonomic and cardiovascular responses associated with ECT may raise concern for tachyarrhythmia and an exaggerated hypertensive response.^14^, ^15^ Accordingly, mECT was undertaken in close collaboration with endocrinology and anesthesiology. In the present case, there were no clinical features suggestive of thyroid storm. Following mECT, agitation and hyperactivity rapidly improved, allowing physical restraint to be discontinued and treatment for Graves' disease to be initiated.

The immediate clinical significance of mECT in this case was the rapid improvement in severe psychomotor agitation and behavioral dysregulation, which restored the feasibility of treating the physical illness. Psychiatric stabilization was, in this sense, a means of enabling necessary medical treatment.

### Limitations and future directions

This report has several limitations. First, it remains unclear whether the psychotic depression was directly attributable to Graves' disease or whether Graves' disease precipitated an endogenous psychiatric episode. Longitudinal observation of thyroid function and psychiatric symptoms, including recurrence after normalization of thyroid function or changes in psychotropic treatment, will be informative. Although no recurrence has been observed to date in the present case, the etiological relationship cannot be established from the current course alone.

Second, because mECT and treatment for hyperthyroidism were administered during overlapping periods, their respective contributions to sustained psychiatric recovery cannot be fully separated. Third, the proposed framework for treatment prioritization is a clinical conceptualization derived from a single case and previous reports, not an established treatment algorithm. Treatment sequencing should be individualized according to psychiatric severity, urgency of the physical illness, medical risk, and the patient's ability to cooperate with treatment.

## CONCLUSION

Psychotic depression occurring in association with Graves' disease may represent either a psychiatric syndrome directly attributable to thyroid dysfunction or an independent mood episode precipitated by the medical illness, and this distinction may remain unresolved during acute treatment. In the present case, severe psychiatric symptoms prevented treatment of Graves' disease. After confirming hemodynamic stability and the absence of clinical features suggestive of thyroid storm, mECT was initiated in consultation with endocrinology and anesthesiology. Rapid improvement in psychomotor agitation and behavioral dysregulation enabled treatment of Graves' disease to proceed. When psychiatric symptoms themselves constitute a barrier to necessary medical care, psychiatric stabilization may appropriately become an initial component of treating the coexisting physical illness.

## GENERATIVE AI STATEMENT

During the preparation of this manuscript, the authors used ChatGPT (OpenAI) to assist with language editing, wording, and organization of the manuscript. All AI‐assisted output was critically reviewed, verified, and revised by the authors, who take full responsibility for the final content. Generative AI was not used for the generation or analysis of clinical data.

## AUTHOR CONTRIBUTIONS

Ayumu Kocha, Aya Akaba, Nozomi Sawasaki, Shinobu Seno, Naoya Hara, and Taro Minato were involved in the patient's clinical care and contributed to drafting the manuscript. Atsuo Nakagawa and Hiroki Kocha supervised the work and contributed to the conceptualization and critical revision of the manuscript. All authors reviewed and approved the final manuscript.

## CONFLICT OF INTEREST STATEMENT

The authors declare no conflicts of interest.

## ETHICS APPROVAL STATEMENT

Ethics approval was not required for this single‐patient case report in accordance with institutional policy.

## PATIENT CONSENT STATEMENT

Written informed consent for publication of the clinical details and anonymized quotations was obtained from the patient.

## CLINICAL TRIAL REGISTRATION

Not applicable.

## Data Availability

Data sharing is not applicable to this article because no research dataset was generated or analyzed for this case report.
